# Phase Evolution of Hybrid Alkali Sulfate-Activated Ground-Granulated Blast Furnace Slag Cements

**DOI:** 10.1021/acssuschemeng.3c05937

**Published:** 2023-12-01

**Authors:** Juan Manuel Etcheverry, Zengliang Yue, Sreejith Krishnan, Yury Andres Villagran-Zaccardi, Philip Van den Heede, Yuvaraj Dhandapani, Susan Andrea Bernal, Nele De Belie

**Affiliations:** † Magnel-Vandepitte Laboratory for Structural Engineering and Building Materials, Ghent University, Technologiepark-Zwijnaarde 60, 9052 Ghent, Belgium; ‡ School of Civil Engineering, University of Leeds, LS2 9JT Leeds, U.K.; § Sustainable Materials, Flemish Institute for Technological Research (VITO), Boeretang 200, 2400 Mol, Belgium

**Keywords:** hybrid alkaline cements, calcium aluminosilicate hydrates, layer double hydroxides, secondary hydration products, alkali-activated slags, quantification of hydration products

## Abstract

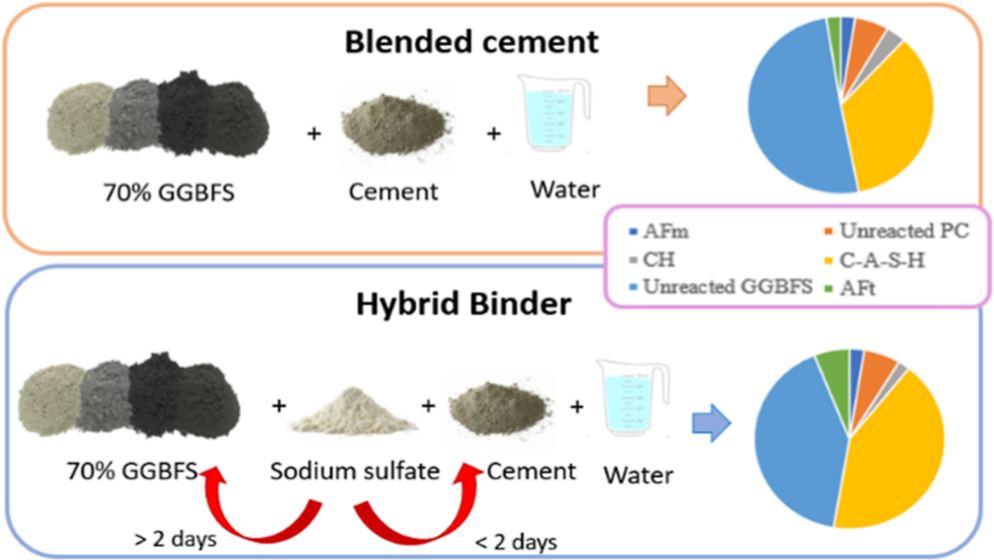

In this study, a hybrid alkali-activated ground-granulated cement consisting of 70% blast furnace slag (GGBFS) and 30% Portland cement (PC) activated with sodium sulfate was studied. Results were compared with those of a blended system without an activator. The addition of the activator significantly increased the kinetics and degree of reaction of these cements, particularly at early curing ages (2 days), without leading to significant changes in the phase assemblage. The main reaction product formed was an aluminum-substituted calcium silicate hydrate (C-A-S-H) type gel, with a Ca/Si ratio comparable to that of the activator-free blended cement; however, in the presence of the activator, sorption of sulfur was observed in the C-A-S-H phase. The formation of secondary phases including ettringite and Ca- or Mg-rich layered double hydroxides was also identified in these cements depending on the curing age and activation addition. This study demonstrates the effectiveness of sodium sulfate in accelerating the phase assemblage evolution in high-GGBFS-content PC-blended cements without leading to significant changes in the reaction products formed, particularly at advanced curing ages. This represents a step forward in the development of cements with a reduced clinker factor.

## Abstract 2

Understanding the phase evolution of hybrid binders will lead to optimized mix designs with a reduced carbon footprint.

## 1 Introduction

The interest in using near-neutral salts, such as sodium sulfate, as an activator for the production of sustainable alkali-activated slag cements is increasing. These activators can be obtained not only from natural sources but also as byproducts from different industrial processes,^1^ offering a feasible and practical solution to implement a circular economy. The activator’s rather low pH makes it safe to handle in comparison to sodium silicates or sodium hydroxide solutions, which historically have been the preferred activators used for the activation of blast furnace slags.

The alkali activation of ground-granulated blast furnace slag (GGBFS) with sodium sulfate and minor addition of Portland cement (PC) has been successfully achieved,^2−4^ with specimens showing very good performance in terms of strength development. A study by Rashad^5^ revealed that the compressive strength of sodium-sulfate-activated slags can be improved by increasing either the slag finesses or the activator dosage. The use of this activator along with a limited amount of PC offers several benefits, including the use of a reduced carbon footprint activator (in comparison to sodium silicates/sodium hydroxides) in activated slag systems, and a contribution to overcome technical challenges associated with the use of ultrahigh contents of blast furnace slags in blended cements such as the regulation of setting times and strength development at early curing ages.^2^ Etcheverry et al.^2^ studied the influence of sodium sulfate dosage on the early age hydration and development of the microstructure of hybrid alkali-activated GGBFS/PC cements. An optimized amount of activator was defined from that study taking into consideration environmental aspects of the cements produced and their performance development. In situ X-ray diffraction (XRD) analysis of such systems revealed a faster consumption of alite and a greater formation of ettringite with the addition of higher activator contents. The interaction between PC and sodium sulfate controls the early age behavior of such cements, while the addition of GGBFS contributes to strength development from 2 days onward. This highlighted the secondary role played by the GGBFS on setting in the hybrid alkali-activated cements studied. Joseph and Cizer^3^ evaluated the influence of the curing temperature on the hydration products and mechanical properties of a commercial CEM III/B with and without sodium sulfate. An increased ettringite formation at all curing ages, along with a densification of hydrates, was reported with the sodium sulfate addition. Fu et al.^4^ proposed a mechanism of hydration for an Australian slag in 50% PC/50% slag-activated systems where the reaction is controlled by the solubility of portlandite as a function of the pH and the Ca activity in the pore solution, which at the same time influences dissolution of the slag. Some progress has been made regarding characterization of the hydration products and phase assemblage of such systems,^1,3,4,6,7^ but this falls short of fully understanding the distinctive chemical features of the reaction products forming in hybrid alkali-activated cements, which is the main focus of the present study.

In hybrid alkali-activated slag (AAS) cements, the content of PC, slag type, and activator dosage are expected to control the type and amount the secondary phases formed, which in turn will determine the performance and durability of the cementitious materials throughout their service life. Bernal^1^ explains that the main binding phase forming in the case of sodium sulfate-activated slags is an alkali aluminum-substituted calcium silicate hydrate (C-(N)-A-S-H), and the secondary phases may vary depending on the activator and the MgO content of the slag under study. For sodium sulfate-activated slags, ettringite (E) is expected to form as the main secondary phase when the content of MgO is low (<8 wt %). Instead, sulfate-bearing layer double hydroxides (LDHs) were observed with high-MgO-content (>13 wt %) slags. The formation of different types of LDHs and the degree of cross-linking of the C-(N)-A-S-H gel in AAS cements have been reported to have a significant impact on durability.^8,9^ A better understanding of the features of this type-gel is still needed to explain the performance of hybrid AAS cement in the hardened state.

In this study, the main reaction products forming in a high volume GGBFS blended cement and a Na_2_SO_4_-activated GGBFS-PC system were investigated following a multitechnique approach applying XRD and thermogravimetry analysis (TGA), along with scanning electron microscopy coupled with energy dispersive X-ray spectroscopy (SEM-EDS). ^29^Si and ^27^Al magic angle spinning (MAS) NMR spectroscopy was applied to determine the structural features of the C-(N)-A-S-H forming in these systems.

## 2 Experimental Methodology

### 2.1 Materials Characterization and Mix Design

A commercial GGBFS and CEM I 52.5 N (PC) were used in this study. Their chemical composition, density, and particle size distribution are listed in Table 1. Particle size distribution was determined by laser diffraction using a Malvern Mastersizer 2000 device (Figure S1-S1a,b, Supporting Information). For the production of the hybrid alkali-activated binder (HB), sodium sulfate (technical grade with a purity >99%) was added as a dry solid (powder) into the mix, dosed at 8% wt per gram of GGBFS. The activator dose used in this study (8 wt % of GGBFS) was identified as the optimum value to maximize the GGBFS dissolution at early ages.^2^ Tap water was used for producing all paste mixes.

**Table 1:** Chemical Composition Determined by X-ray Fluorescence Analysis and Physical Properties of the PC and GGBFS Used

	PC (CEM I)	GGBFS
Chemical Composition [% m/m]		
CaO	64.30	40.80
SiO_2_	18.30	33.30
MgO	1.40	7.84
Al_2_O_3_	5.20	12.30
Fe_2_O_3_	4.00	0.39
Mn_2_O_3_		0.36
Cl	0.06	
BaO		0.31
SO_3_	3.50	2.30
Na_2_O	0.32	0.44
K_2_O	0.43	0.67
TiO_2_		2.30
LOI (lost on ignition)^a^	2.30	0.01
insoluble residue	0.40	
CaO + MgO + SiO_2_	84.00	81.94
(CaO + MgO)/SiO_2_	3.59	1.46
Physical Properties		
particle size distribution (μm) d10/d50/d90	2.3/10.8/29.4	1.3/7.6/26.8
density [kg/m^3^]^b^	3160	2890

a LOI determined according to EN 196-2.

b Determined according to EN 196-6.

Pastes were produced with a 70/30 GGBFS/PC weight ratio and a 0.45 water/binder (w/b) ratio. This was selected based on the fact that 30% is the maximum relative content of PC typically used in the production of hybrid alkali-activated systems including other supplementary cementitious materials (e.g., FA).^10,11^ This was with the aim of ensuring certain early age strength and a sufficient portlandite reserve (provided by the PC hydration) to favor supplementary cementitious materials’ (SCMs’) reaction at later ages.

The mix design of the studied pastes is presented in Table 2. For replacement levels below 70%, the addition of an activator is usually not required to achieve an acceptable compressive strength level at 2 days of curing, as is the case for CEM-III/B.

**Table 2:** Mix Proportions of the Studied Pastes

sample ID	GGBFS/binder	PC/binder	water/binder^a^	Na_2_SO_4_ (% wt GGBFS)
SS0	0.70	0.30	0.45	0
SS8	0.70	0.30	0.45	8

a “Binder” refers to the combination GGBFS + PC.

Materials were preconditioned for 24 h in a room at 20 °C and (60 ± 5)% relative humidity before mixing. Pastes were produced by hand mixing for 2 min. Cylindrical molds (Ø: 25 mm and h: 100 mm) were filled in and sealed with a plastic cap. Samples were demolded after 24 h and cured in a wet-room (20 °C and >95% relative humidity) until testing.

At ages of 1, 2, 7, 28, and 90 days, 3 to 5 mm thick disks were sawn from the cylinders, and the hydration was stopped by two cycles of immersion in isopropanol as described in refs (12) and (13). For XRD and TGA analyses, the samples were ground to powder before hydration stoppage; for SEM analysis, the discs were treated directly with the isopropanol. Each cycle lasted 15 min for the powdered samples and 1 h for the whole discs, during which the samples were continuously spinning. Samples were washed by diethyl ether and dried at 40 °C for (8 ± 2) min and then placed in a vacuum desiccator until testing. Such a period of time was never longer than a week. After this treatment, for XRD and TGA analysis, selected samples were ground (when necessary) again using a mortar and pestle and sieved so that all the material passed a 65 μm mesh, whereas separate whole discs were impregnated with epoxy for SEM observations.

### 2.2 Testing Methods

- Powdered samples were used for XRD analysis. As an internal standard, 10% wt of ZnO was utilized, with the aim of conducting Rietveld analysis for quantifying the hydration products forming. The XRD measurements were performed in a Bruker D8 device equipped with an Euler-cradle, Cu radiation tube, and LYNXEYE XE-T detector (filament length—12; sample length—20; receiving slit length—16; primary soller angle—2.5°; secondary soller angle—2.5°; axial n beta—30. Fixed slits settings—0–6 mm; minimum angular range—5–70° 2θ Cu Kα; step size—0.02° 2θ; counting time—1 s/step; power—40 kV; current—30 mA; side loading of samples). Quantitative XRD was obtained by Rietveld and PONKCS methods utilizing TOPAS (Academic) V7 software. Additional information concerning the refinement and phase information can be found in the Supporting Information file.
- TGA was performed in a Netzsch STA 449 Jupiter TGA-DTA Analyzer. The temperature ranged from 20 to 1100 °C at a heating rate of 10 °C/min in an inert nitrogen atmosphere. About 50 mg of sample was used in TGA experiments. The tangential method described in ref (13) was adopted for the quantification of portlandite from DTG curves.
- The SEM samples were stored under vacuum for 1 day after the hydration stoppage and then impregnated with epoxy in a vacuum chamber (resin-to-hardener ratio was 3:20 in wt). Samples were polished using 500 to 2000 grade SiC papers and 3 to 1 μm diamond paste. Samples were carbon-coated with a 30 nm layer (carbon rods). The SEM-BSE experiments were conducted in a Jeol JSM-7600F field emission scanning electron microscope, with an in-lens Schottky electron source. At least 30 SEM-BSE micrographs were evaluated to obtain the data in Figures 7 and 8. Additional information about SEM-BSE images and quantification of phases from these images is found in the Supporting Information file.
- The same samples were used for EDS analysis, using a Zeiss Evo 15 scanning electron microscope operating at 20 kV at working distance of 8.5 mm, coupled with an Oxford Instruments X-Max 150 EDS detector. Over 100 EDS points (20 s per point) were collected in the outer reaction product region to determine its chemical composition. All results are presented in atomic percentages.
- Solid-state ^27^Al and ^29^Si MAS NMR were conducted in a Bruker Avance III HD spectrometer with a 400 MHz wide bore magnet (magnetic field 9.4 T). For ^29^Si MAS NMR, the frequency of operation was 79.5 MHz. A 7 mm zirconia rotor and a spinning speed of 6 kHz were utilized. The duration of pulse was 5.5 μs at 90° with a relaxation delay of 40 s. 4096 scans were collected for each sample. For ^27^Al MAS NMR, the frequency of operation was 104.3 MHz. A zirconia rotor and spinning speed of 12 kHz in a 2.5 mm solid-state MAS probe was utilized. The duration of pulse was 0.23 μs at 90° with a relaxation delay of 0.5 s. 16,384 scans were collected for each sample. ^29^Si and ^27^Al chemical shifts were referenced to external samples of tetramethylsilane (TMS) and yttrium aluminum garnet (YAG), respectively, the latter with the hexa-coordinated site referenced to 0.7 ppm. Signals were normalized by area. Additional information can be found in the Supporting Information file.

**Figure 1:**
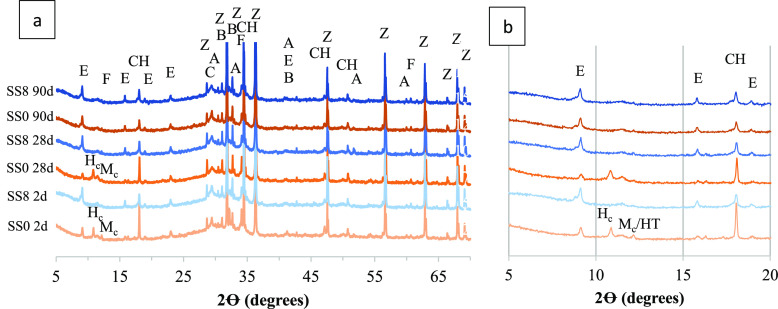
XRD patterns of (a) 2, 28, and 90 days for SS0 and SS8. (b) Zoom-in of the 5 to 20° 2θ Cu Kα region. Ettringite (E), portlandite (CH), calcite (C), hemicarbonate (Hc), hydrotalcite (HT), monocarbonate (Mc), zincite (Z), alite (A), belite (B), and ferrite (F).

**Figure 2:**
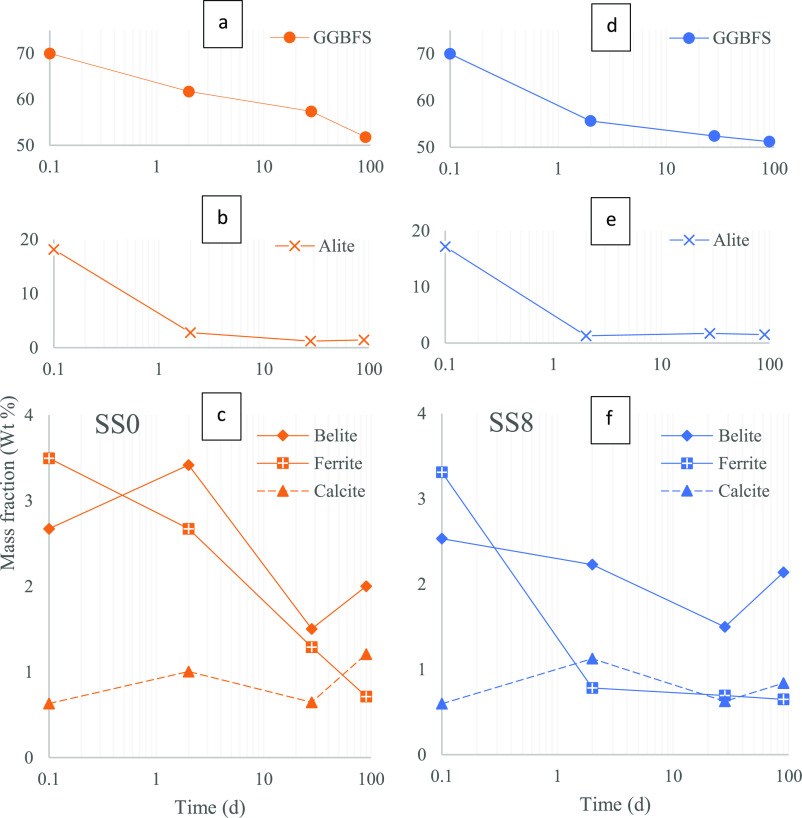
Mass fraction (%) of GGBFS and clinker phases in SS0 (a,c) and SS8 (d,f) pastes as a function of the curing time determined by QXRD analysis.

**Figure 3:**
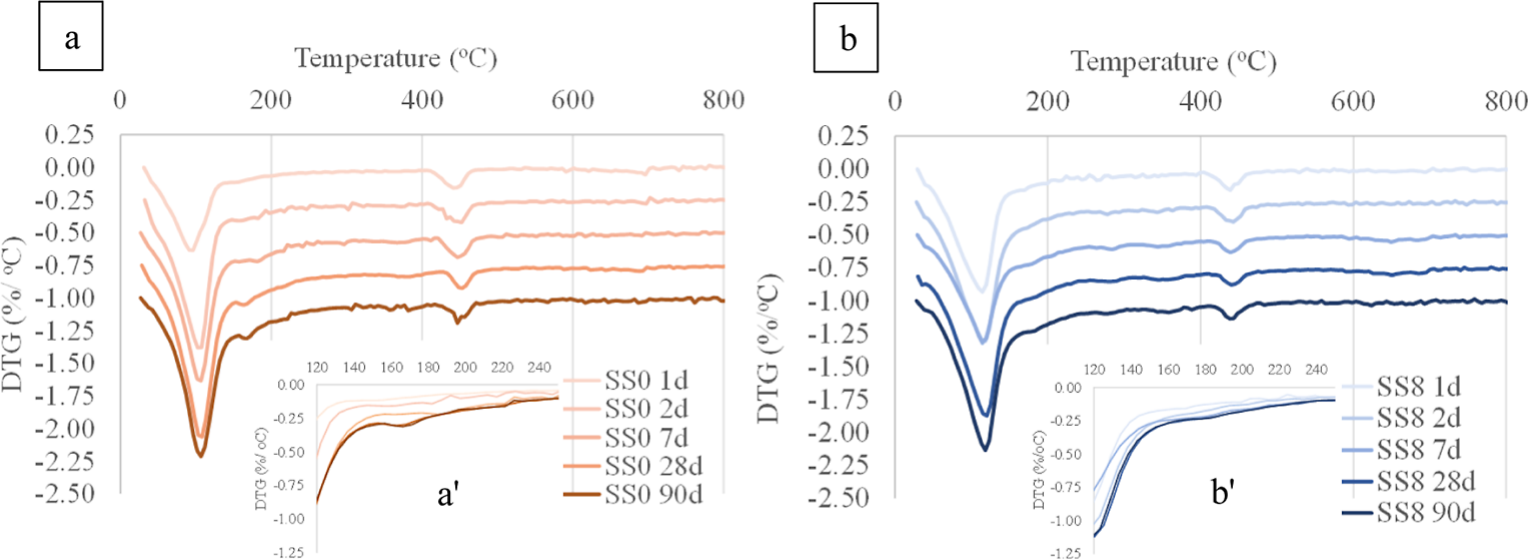
DTG profiles at 1, 2, 7, 28, and 90 days for SS0 (a) and SS8 (b). Zoom-in DTG profiles in the range of 120–250 °C (a′,b′). Vertical offset between subsequent curves is always 0.25%.

**Figure 4:**
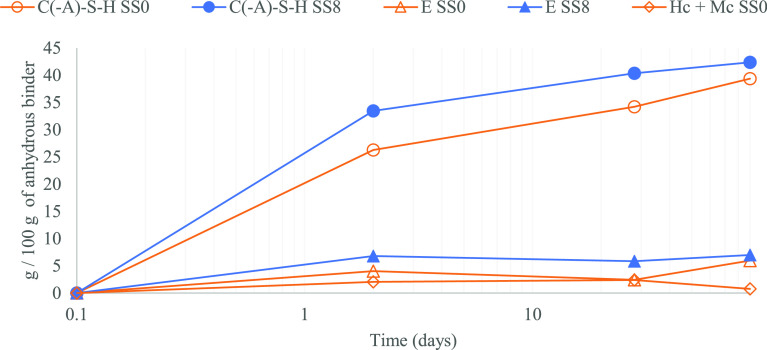
C(-A)-S-H, ettringite (E) and AFm phases evolution over time for SS0 (empty orange markers) and SS8 (filled blue markers).

**Figure 5:**
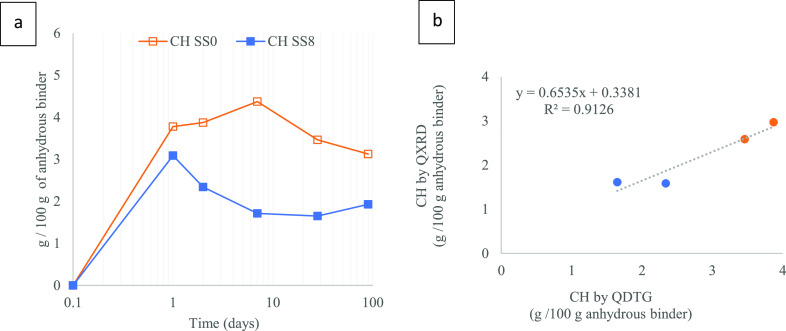
CH contents in pastes: (a) evolution of CH (TGA) for SS0 and SS8 and (b) comparison CH quantification between 2 and 28 days of hydration (g/100 g of anhydrous binder) from XRD and TGA.

**Figure 6:**
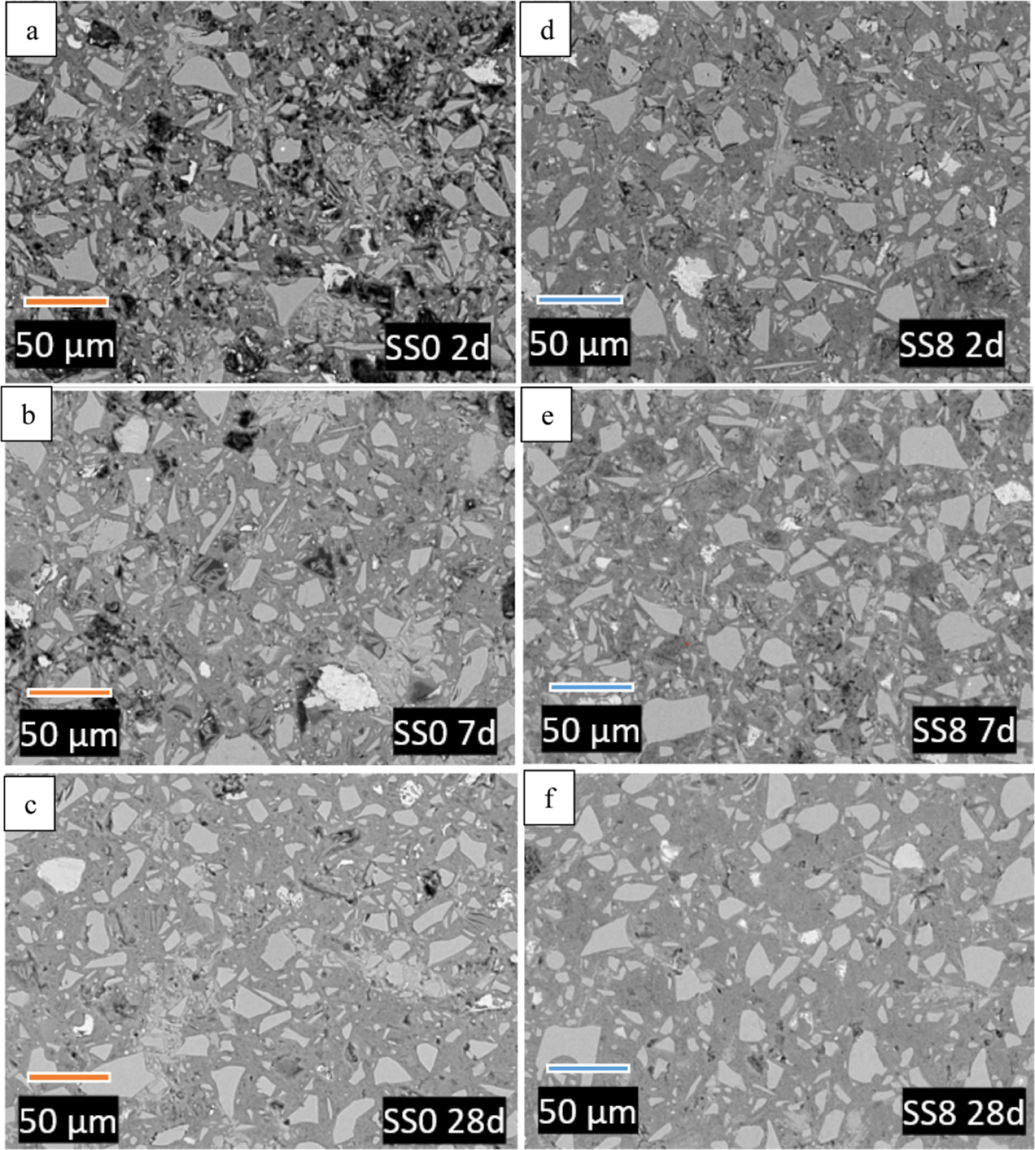
BSE images of paste samples containing 0 (a–c) and 8 wt % (d–f) sodium sulfate as a function of curing time.

**Figure 7:**
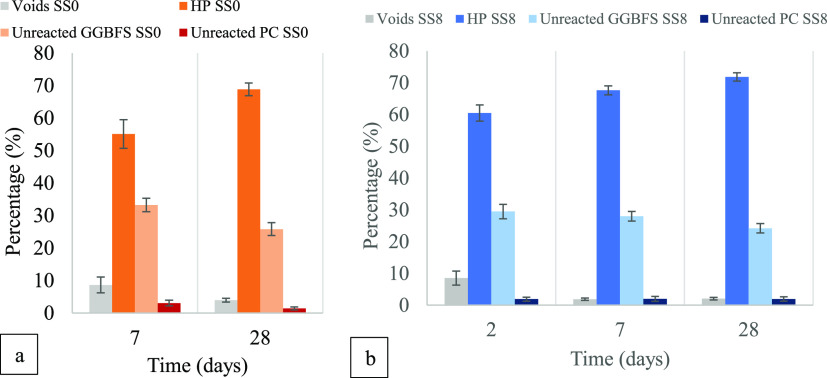
Quantification of SEM-BSE images at ages of 2, 7, and 28 days for samples containing 0 (a) and 8 wt % (b) sodium sulfate. The quantification was based on the grayscale analysis of BSE images as observed in Figures S1–S4 in the Supporting Information file. HP stands for hydration products.

**Figure 8:**
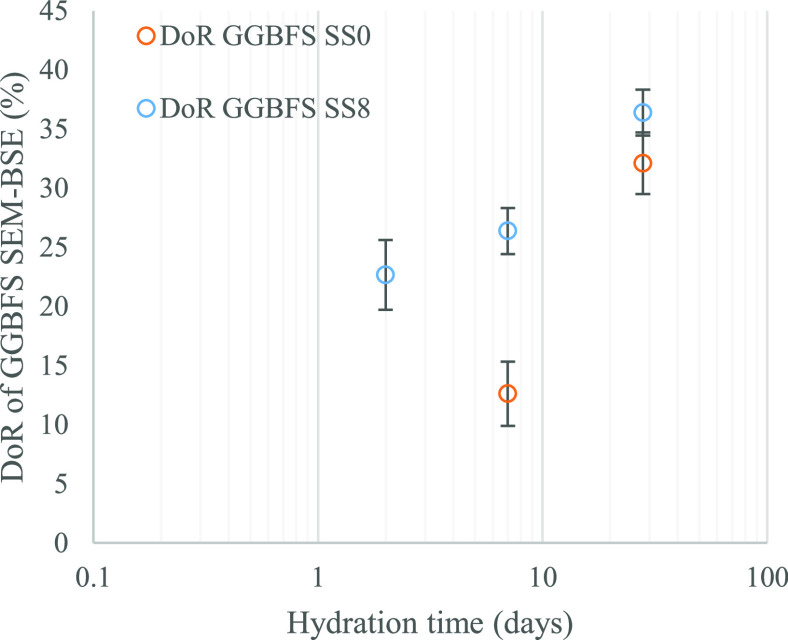
GGBFS DoR over the time of curing for SS0 and SS8 systems.

## 3 Results and Discussion

### 3.1 Phase Assemblages

The XRD patterns for SS0 and SS8 at 2 and 28 days of hydration are plotted in Figure 1a. A zoomed-in view of the 5 to 20° 2θ Cu Kα region is plotted in Figure 1b. Ettringite is identified as one of the main hydration products and the noncrystalline phase (quantified with the aid of the internal standard) that does not match the pattern of the raw GGBFS (i.e., unreacted GGBFS) is associated with a significant presence of an aluminum-substituted calcium silicate hydrate (C(-A)-S-H). Minor contents of hemicarbonate (Hc) and monocarboaluminate (Mc) are also observed for the SS0 system. The CEM I utilized in this study contained limestone (2.1 wt % of calcite was identified from QXRD analysis), and this can explain the formation of minor amounts of Hc or Mc in the blended system.^14^

Figure 2 shows the mass fraction of GGBFS and clinker phases determined by QXRD in SS0 Figure 2a–c and SS8 Figure 2d–f pastes as a function of time. The amount of clinker phases such as alite and ferrite is reduced as reaction progresses in the presence of sodium sulfate, in agreement with the results presented by,^15^ which explain in further detail how the combination of sulfates and alkalis influence the growing of C(-A)-S-H. Aluminate reaction is also accelerated in hybrid alkali-activated cements,^2^ consistent with the results of this study. Such an accelerating trend is not so clear for belite as negligible differences are observed in the cements independently of the activator content. The evolution in the hydration degree of these phases and reaction of GGBFS (Figure 1) is in agreement with previous studies that separately addressed the acceleration of alite,^15^ PC,^16^ and GGBFS^5^ reaction in slag Portland blended cement with sodium sulfate addition. Although Figure 2 depicts an increase in the GGBFS degree of reaction (DoR) due to the addition of the activator, the amount of unreacted GGBFS seems to remain similar to or without the activator at 90 days.

### 3.2 Influence of Sodium Sulfate on the Main Hydration Products

DTG curves resulting from the TGA analysis are plotted in Figure 3a,b, for SS0 and SS8 pastes, respectively. For both systems, two main peaks are clearly noted: one located between 50 and 200 °C, associated with the presence of ettringite and C-S-H or C(-A)-S-H products;^13^ and a second less intense from 400 to 500 °C, associated with the decomposition of portlandite.^13^

The first peak progressively increases as the curing time increases, which is consistent with higher degrees of hydration. This is observed in both blended cements with or without sodium sulfate addition. An important observation is the more significant shift of this first peak from 1 to 2 days for SS8 in comparison with SS0, revealing an important acceleration in the early reaction and microstructure development when sodium sulfate is added to GGBFS/PC blended cements. This is also supported by broader first peaks with a similar intensity (mainly associated with presence of C-S-H/C-A-S-H and ettringite) for the hybrid alkali-activated system SS8, suggesting a greater amount of these hydrates forming at a given curing age compared with the SS0 mix.

The quantification of the main hydration products obtained by QXRD (g/100 g of anhydrous binder) is shown in Figures 4 and 5. A significant difference is observed in terms of C(-A)-S-H formation, which develops faster for SS8 than for SS0 even after 2 days of hydration (Figure 4). The amount of C(-A)-S-H in the systems increases similarly for both systems from 2 to 7 days but it remains higher in SS8 due to the initial difference (<2 days). Although the rate of C(-A)-S-H precipitation increases between 7 and 28 days for SS0 compared to SS8, at 28 days of hydration, the SS8 system still presents a higher in C(-A)-S-H content than the blended system. These results are in good agreement with the higher degree of hydration of GGBFS (Figure 2a,d) and alite (Figure 2b,e) identified by QXRD and the DTG results (Figure 3). In addition, Figure 4 reveals ettringite values of 4.0 wt % (2 days) and 2.4 wt % (28 days) for SS0, and 6.8 wt % (2 days) and 5.8 wt % (28 days) for SS8, which is also consistent with the DTG observations.

Joseph and Cizer^3^ reported much higher ettringite contents (>10% wt.) when a commercial CEM III B (additional amount of gypsum) was activated with sodium sulfate. Instead, Fu, et al.^4^ measured (2 days) similar ettringite contents to the ones in the present study. Such different values seem to be explained by the differences in sulfate concentrations in the pore solution, as sodium sulfate is more soluble than gypsum. A recent paper^17^ explains that although elevated sulfate concentrations (in solution) accelerates the formation of C-S-H, it also hinders ettringite precipitation. A previous study by some of the authors^2^ appears to indicate that sulfates consumed at very early age are also being provided by gypsum before the acceleration/deceleration peak detected by isothermal calorimetry. The absorption of sulfate on the C_3_A^18^ and mainly C(-A)-S-H phases^19^ is reported as the controlling mechanism. Nevertheless, it is worth pointing out the influence of the curing temperature on the evolution of these Al-rich phases^20^ where factors such as the pretreatment of the sample may lead to significant variations in the content of ettringite as well.^3,13^ These make the comparison among different studies challenging, and in hybrid alkali-activated cements where both gypsum and sodium sulfate are present, further studies are necessary to elucidate the effect of the different sulfate sources at different stages of reaction depending on the dosage of the activator.

As hydration progressed, a shoulder around 170 °C was identified for the blended system (DTG, Figure 3a′), assigned to the formation of monosulfoaluminate (Ms)^20^ or monocarboaluminate (Mc).^13^ Formation of AFm phases is reduced when sodium sulfate is added (DTG, Figure 3b′), instead more ettringite is observed in such a case (Figure 4, QXRD), in agreement with ref (21), which proposed that bulk SO_3_/Al_2_O_3_ and CO_3_/Al_2_O_3_ ratios control the combination of AFm and AFt phase assemblage found in cementitious systems. Mass balance calculations conducted according to ref (22) for the cementitious systems evaluated in this study predict the formation of Ms, Hc, and E for SS0 and mostly E for SS8 (Figure S1-S2, Supporting Information). Ms is not clearly observed by QXRD (Figure 1) and the possible reasons for Ms not being observed by XRD are discussed in Section 3.5. In addition, minor peaks at 180 and 350 °C were noticed after curing times of 28 days in SS8. According to previous research in similar systems,^23,24^ this might be associated with strätlingite, which is also difficult to identify by XRD due to its poor crystallinity.^24^

Figure 5a shows the evolution of portlandite contents (TGA) over curing time quantified following a similar procedure described in ref (13). These values are compared to those calculated by QXRD (Figure 5b). Both techniques consistently measure a similar trend and values of portlandite obtained from QXRD are similar to those obtained from TGA.

Portlandite is formed upon hydration of C_2_S (1.91/100 g cement in this study) and C_3_S (29.4/100 g cement in this study). The quantities of belite and alite correspond to the percentage of these phases obtained from QXRD of the anhydrous cement (Table S1-S2, Supporting Information), which leads to a theoretical maximum amount of portlandite formation of 9.4/100 g binder. However, this value considers 100% of PC hydration, which is clearly not happening at 1 day of curing. In a previous work,^2^ the reaction degree of 30% PC + 70% quartz (with and without the same amount of sodium sulfate) during the first week of hydration was calculated, revealing a PC reaction degree (1 day) of 40% without the activator and 64% with sodium sulfate. For such a DoR, the maximum portlandite content would be 3.8/100 g of anhydrous binder for SS0 and 6/100 g of anhydrous binder for SS8, only considering the acceleration of the PC reaction provided by the activator. For the SS0 system, the theoretical value is in very good agreement with the one measured by TGA at 1 day of hydration (3.7/100 g of anhydrous binder). It suggests that due to the sodium sulfate addition, the portlandite content in SS8 (3.0/100 g of anhydrous binder at 1 day of hydration) is significantly lower than the theoretical value.

The reaction between sodium sulfate and portlandite to form gypsum and sodium hydroxide used to be pointed out as the mechanism rising the pH and increasing the dissolution of the SCMs.^25−27^ According to,^4^ although the addition of sulfates takes Ca out from the pore solution, no gypsum is being formed. The pH is increased through a more complex mechanism involving Ca activity in the pore solution, which is controlled by the solubility of portlandite, while there are yet enough sulfates to prevent the precipitation of Al-rich phases (early hydration). After the sulfate depletion point, the pH and a decreased calcium activity in the pore solution control the dissolution of GGBFS.^4^ For the SS0 system, the portlandite content is increased up to 7 days, in agreement with a slower reaction of PC, a period after which portlandite is slightly consumed. Instead, very limited variation in the content of portlandite is measured after the second day of hydration for SS8. Nevertheless, the dissolution and reaction of GGBFS continues.

This mechanism also explains why the addition of portlandite powder in minor quantities enhances the early age dissolution of GGBFS, but the further dosage increase has almost no effect in the reaction kinetics.^28^ These are interesting results because the sole analysis that mixing sodium sulfate and PC reduces the portlandite formation could lead to a misleading prediction of a lower DoR of the GGBFS. In reality, the sole increase in the pH in the pore solution will have a more beneficial effect in terms of GGBFS dissolution. In addition, measuring the reactivity of Ca-rich SCMs by portlandite consumption is not expected to provide any representative result in hybrid alkali-activated systems with sodium sulfate as the activator. This is consistent with^29^ where the limited consumption of portlandite in blended cements was also highlighted.

### 3.3 Effect of Sodium Sulfate Addition on Microstructure Development of High-GGBFS-Content–PC Blends

Figure 6 shows the SEM-BSE grayscale images for the blended and the hybrid alkali-sulfate activated binder as a function of the curing time. Particles of GGBFS can be identified by their angular shape and homogeneous tone of gray. Unreacted PC particles are very rich in calcium (Ca) and generally contain very low amounts of Mg and Al (which makes them brighter than GGBFS), some of them are rich in Fe, which can lead to a brighter shade (almost white). PC particles may show some surface roughness due to the very low “migration” of Fe in alkaline solutions. The CH-rich zone is brighter than C-(A-)S-H zones. The dark (almost black) part of the image is the porosity filled in with the epoxy resin (Figure S1-S4, Supporting Information).

Figure 6 shows a clear reduction in porosity, together with a greater PC and GGBFS reaction, as discussed in previous sections. Blended system (Figure 6a–c) and hybrid system (Figure 6d–f). A more homogeneous and less porous microstructure is observed in the SS8 after 2 days of curing (Figure 6a) compared to SS0 2d (Figure 6d), consistent with the increased DoR at an early age for the sodium sulfate-containing system, in agreement with QXRD (Figure 4) and TGA results (Figure 3).

Figure 7 shows the quantifications of the porosity, reaction products, unreacted PC, and GGBFS particles derived from the analysis of BSE images. Reliable measurements of porosity and unreacted particles for SS0 at 2 days of hydration were not possible due to the weakness of the cementitious matrix for it to be properly polished.

On the one hand, there is a progressive reduction in porosity for the blended system; 8.6% (7 days) to 3.9% (28 days), while for the hybrid alkali-activated system, the porosity is 8.5% at 2 days and it reduces sharply to 2% already at 7 days, without further significant reduction at 28 days. The reduction in porosity is a consequence of the formation of reaction products discussed in previous sections, with more reaction products being related to a lower porosity.

The development of microstructure is slower for SS0 than for SS8, which is explained by the low amount of PC in the system, which makes it very dependent on the contribution of GGBFS reaction at very early ages.^33^ The amount of unreacted PC in the SS0 system was revealed to progressively reduce up to 7 days by,^2^ remaining relatively stable from 7 to 28 days, as is the case in the present study as well. By the addition of sodium sulfate, the unreacted PC detected by image analysis reduces much faster during the first 2 days and then it remains fairly the same up to 28 days, showing already at 2 days a similar hydration degree to the one observed for SS0 at 7 days (<3% of anhydrous cement in Figure 7). This confirms the important acceleration in the PC reaction at a very early age, normally associated with the interaction alite/sodium sulfate,^14^ with the GGBFS playing a secondary role up to 1 day of hydration.^2^ A faster formation of C(-A)-S-H has been discussed above (Figure 4) and explained by ref (2) using in situ XRD, which also revealed a greater formation of ettringite and monosulfate in the hybrid system between the first and third days of hydration.

The addition of sodium sulfate in SS8 induced the following changes in the cements evaluated: (1) GGBFS achieves a higher reaction degree (observed by the relative reduction in the area of unreacted GGBFS) at 2 days compared to SS0 at 7 days; (2) the hydration products in SS8 at 7 days present a comparable relative volume to that of hydration products in SS0 at 28 days; (3) the amount of hydration products in SS8 at 28 days remains higher than for SS0, suggesting a greater reaction degree of the GGBFS.

The calculation of the GGBFS DoR from the BSE image analysis is performed using eq 1.^30^
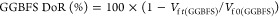
1where *V*_f 0(GGBFS)_ is the volumetric fraction of GGBFS at time zero and *V*_f *t*(GGBFS)_ is the fraction of GGBFS at a certain hydration time (*t*).

Figure 8 shows the GGBFS DoR over time for the SS0 and SS8 systems. Measurements at 2 days were not possible for the SS0 system due to inconveniences during sample preparation related to the weakness of the cementitious matrix (particles of GGBFS were partially removed from the surface during polishing). The trend observed for SS0 seems coherent, with GGBFS DoR increasing from 12% at 7 d to 32% at 28 d. The reacted GGBFS progressively increases from 2 to 28 days for SS8, with the advantage that about 20% of GGBFS has already reacted within 2 days. Moreover, the GGBFS DoR is always higher for SS8 than for SS0 at similar hydration times (7 and 28 days). This is a consequence of the activator addition, increasing pH, and removing Ca from the pore solution, which fosters further GGBFS dissolution. These values are consistent with the formation of hydration products (Figure 7) discussed in this section and in Section 3.2 (Figure 4) as well.

### 3.4 Characterization of C(-A)-S-H Type Gel and Secondary Reaction Products

**Figure 9:**
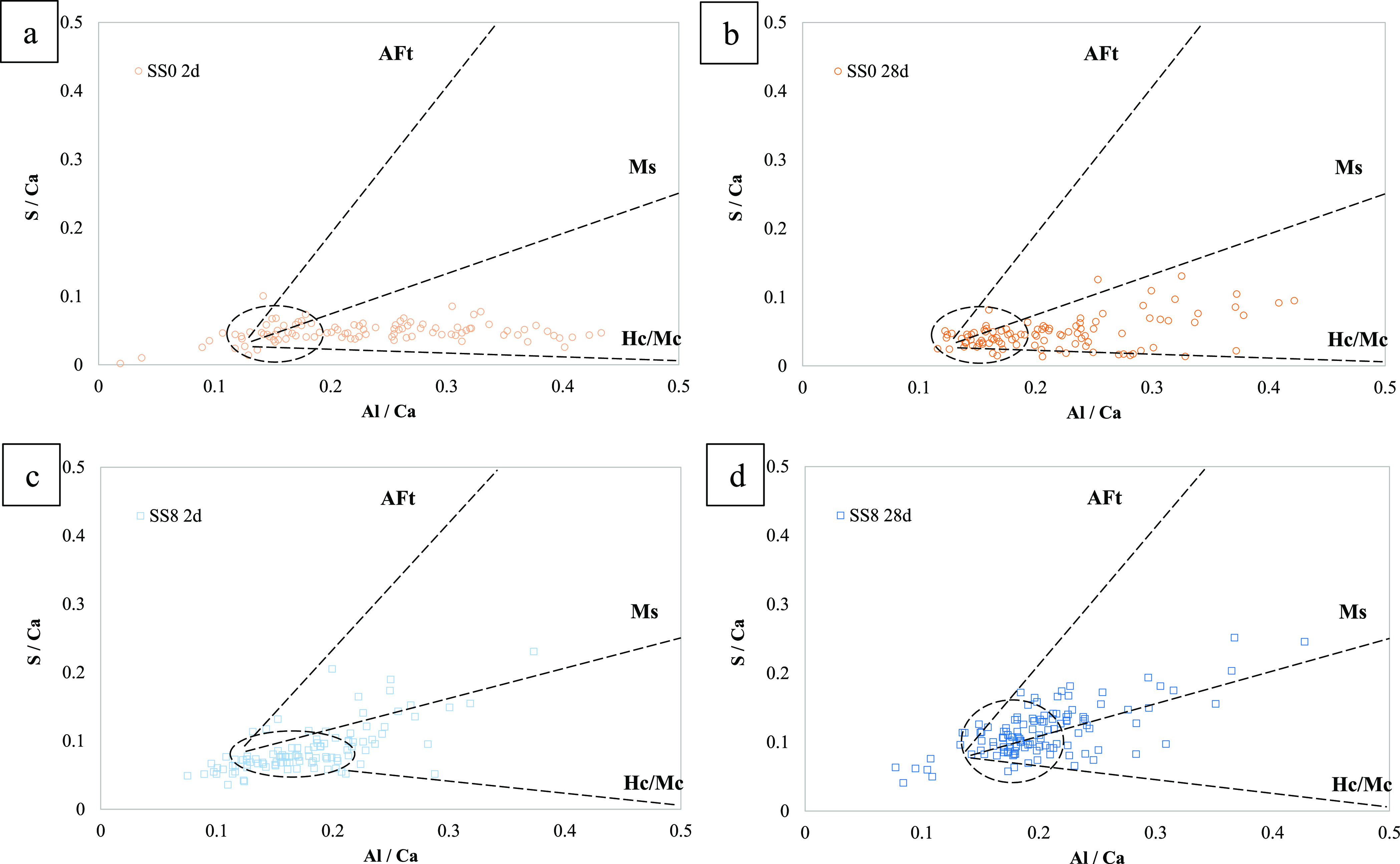
S/Ca versus Al/Ca atomic ratios of SS0 (a,b) and SS8 (c,d) hydrated for 2 (a,c) and 28 days (b,d).

Figure 9 shows the S/Ca versus Al/Ca atomic ratios resulting from SEM-EDS point analysis of SS0 Figure 9a,b and SS8 Figure 9c,d hydrated for 2 and 28 days. In the blended cements, the presence of Hc at 2 days is in agreement with the results of QXRD shown in Section 3.1, and the combined presence of Hc and Ms correlates well with the peak at 170 °C noticed by TGA at advanced hydration times (Figure 3a′). For the SS8 system, a highly intermixed C-A-S-H and sulfate is observed even at 2 days of curing (Figure 9). Such intermixing is maintained at 28 days of hydration and the cloud of points remains with a higher content of sulfates (S/Ca = 0.10) than for the blended system (S/Ca = 0.05), consistent with the addition of sodium sulfate to these systems.

**Figure 10:**
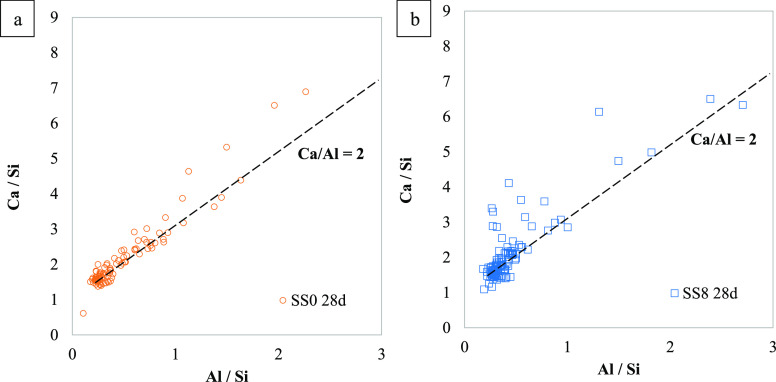
Cal/Si versus Al/Si atomic ratios of SS0 (a) and SS8 (b) hydrated for 28 days. The dashed line indicates that Ca/Al = 2.

Figure 10 plots the Ca/Si versus Al/Si atomic ratios of SS0 Figure 10a and SS8 Figure 10b hydrated for 28 days, which suggests the presence of C(-A)-S-H gel in both systems. The dashed line in Figure 10 showing the ratios Ca/Al = 2 is given to clarify the existence of AFm phases, regardless of the sodium sulfate addition. A similar content of Al in the C(-A)-S-H is observed for both systems with fairly similar Ca/Si atomic ratios (∼1.5) as well.

The incorporation of Mg in C-S-H in CEMII/B activated with sodium sulfate was suggested in a previous study,^3^ which is unlikely to happen because of the limited formation of a solid solutions between C-S-H and M-S-H.^31^ The presence of M-S-H (a very unstable phase) is not observed in either SS0 nor SS8. Such a statement is confirmed by Figure 13 (Section 3.5 below), where no band is visible in the ^29^Si MAS NMR spectra between −90 and −100 ppm (a characteristic peak for M-S-H due to the Q3 silica in tetrahedral coordination^31^).

**Figure 11:**
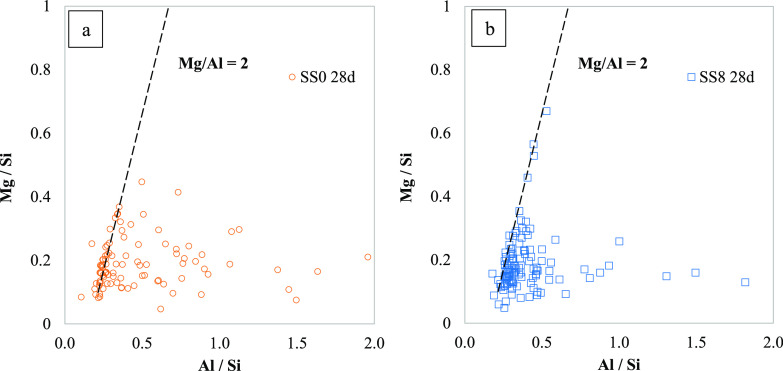
Atomic ratios calculated from EDS data for PC-GGBFS blends with (a) 0 and (b) 8% wt sodium sulfate after 28 days of curing plotted as Mg/Si vs Al/Si. The dashed line indicates the Mg/Al = 2 where the Mg-/Al-rich LDHs would be expected.

**Figure 12:**
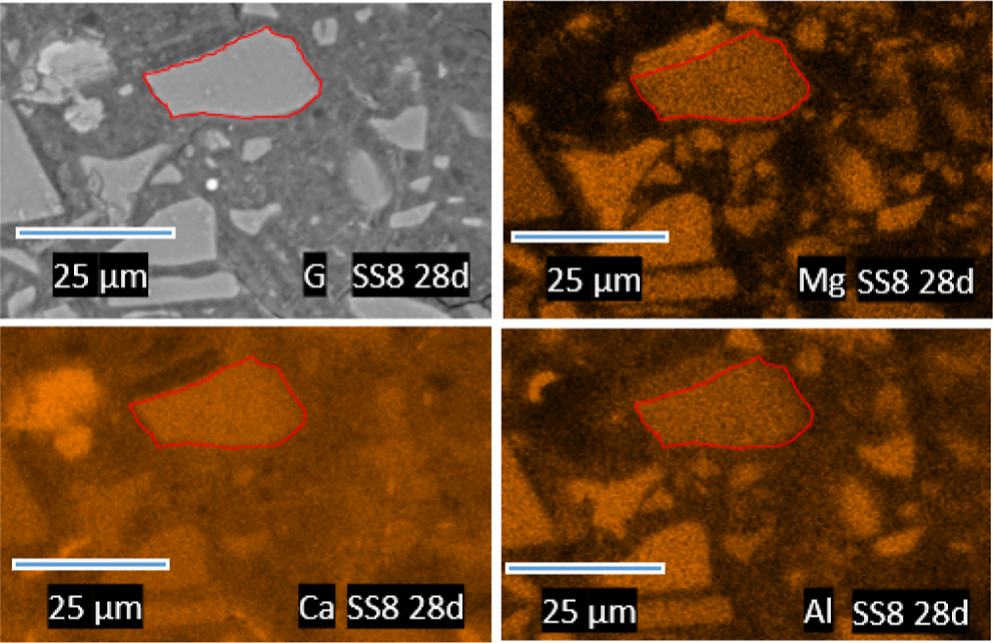
SEM-BSE image (G) of SS8 hydrated for 28 days and its respective Mg, Ca, and Al elemental mappings. The red outline indicates the shape seen in the SEM-BSE image.

**Figure 13:**
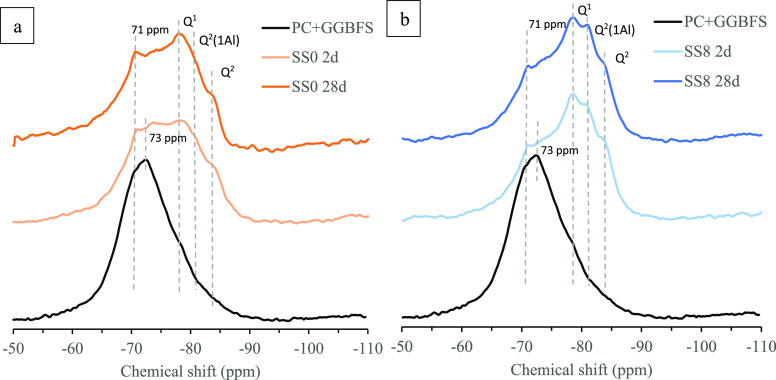
^29^Si MAS NMR spectra after 2 and 28 days of hydration for the SS0 system (a) and SS8 system (b). A third curve corresponding to the PC/GGBFS anhydrous blended sample is included as well.

The GGBFS in this study has a medium content of MgO (∼8%), thus participation of Mg in the reaction is as expected. Figure 11 suggests that a limited amount of Mg-/Al-rich LDHs intermixed in the outer C-A-S-H forms in these cements. The dashed line in Figure 11 showing the Mg/Al ratios of 2 is given to clarify the existence of hydrotalcite-like phases. It is well-known that Mg-rich phases precipitate close to the unreacted GGBFS particles in both blended^32^ and AAS cement.^33^ Therefore, if SEM-EDS points are taken in the outer C-S-H (as was the case in this study), a limited amount of Mg would be expected.^3,4^ Although rims of Mg are not seen in SEM-BSE images at the curing ages evaluated in this study, Mg, Ca, and Al mappings show zones within the “unreacted” GGBFS particles with a reduced amount of Ca but higher intensities of Mg and Al (Figure 12). Such mappings seem to confirm Mg/Al LDHs highly intermixed with the C-S-H. The volumetric amount appears to be very low in comparison to other hydrates since this is observable next to some GGBFS particles only. These zones are identified for both SS0 and SS8. In addition, a larger (but still low) amount of hydrotalcite (in the order of 1.5 g/100 g anhydrous binder) was reported as hydration progresses.^3^

### 3.5 NMR Spectroscopy

The ^27^Si MAS NMR spectra are shown in Figure 13. The GGBFS-PC anhydrous blend is identified with a broad peak (fwhm 20 ppm) centered at −73 ppm. Separated measurements on anhydrous PC and GGBFS powders reveal resonances at −71 ppm (assigned to belite + alite) and −73 ppm (assigned to alite) for the PC^34^ and a single band centered at −73 for the GGBFS (Figure S1-S5, Supporting Information). The PC sharp resonance centered at −71 ppm is the result of belite overlapping with a broader alite feature. Different alite/belite ratios may induce a different shape of the ^27^Si MAS NMR spectra.^34^

Figure 13 shows the ^29^Si MAS NMR spectra at 2 and 28 days for the SS0 (Figure 13a) and SS8 (Figure 13b) systems, respectively. In addition to the bands associated with the unreacted PC/GGBFS blend, three more bands can be identified. In the cements literature,^35,36^ the bands at −78, −81, and −84 ppm are normally attributed to Q^1^, Q^2^(1Al), and Q^2^ silicate sites. For the blended system in the absence of sodium sulfate (SS0), Q^1^ and Q^2^ resonances are identified at 2 days. An overall increase in the intensity of these two bands is noticed, followed by a reduction in the intensity of the resonances in the region where the anhydrous blended was observed. Q^2^(1Al) becomes visibly less abundant than in SS8.

Conversely, three bands are clearly visible for the hybrid system from 2 days of curing. A limited variation in the intensity of such bands is observed between 2 and 28 days of hydration. A third band is found between the aforementioned ones. This band (−81 ppm) is associated with Q^2^(1Al) and suggests that the C-S-H chains are incorporating Al in a binding and/or pairing position, consistent with EDS results in Figure 9. Al in cross-linked sites is noted neither for SS0 nor for the SS8 system; i.e., Al substitutions in tetrahedral sites were clearly detected in Q^2^(1Al) sites for the SS8 system.

**Figure 14:**
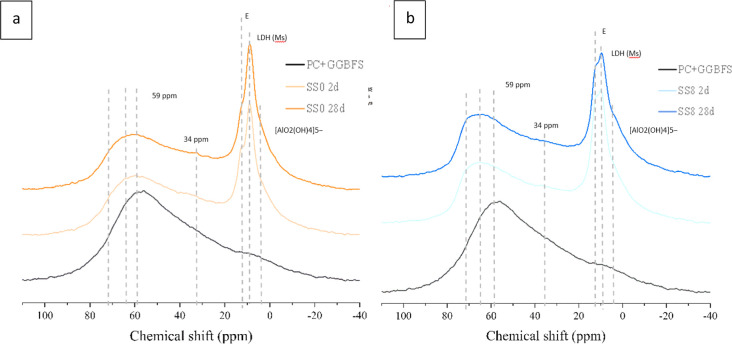
^27^Al MAS NMR spectra after 2 and 28 days of hydration for the SS0 system (a) and SS8 system (b). A third curve corresponding to the PC/GGBFS anhydrous blended is included as well.

In Figure 14, ^27^Al MAS NMR spectroscopy reveals the presence of three distinct aluminum environments. The spectrum of the unreacted GGBFS-PC blend presents a broad band centered at 59 ppm, indicating the presence of aluminum on a tetrahedral site inside the anhydrous GGBFS.^37^ The bands within the range of 50–80 ppm are linked to Al(IV) and are assigned to the tetrahedral aluminum environments present in the C(-A)-S-H gel.^38^ Conversely, the aluminum sites with chemical shift values below 20 ppm are attributed to Al(VI) resonances and associated with the aluminum sites found in ettringite and LDH structures (Ca/Al and Mg/Al).

Although constituent peaks in the tetrahedral aluminum environment are difficult to distinguish, some features are highlighted. Pardal et al.^39^ reported that differences in the slope of the ^27^Al MAS NMR spectra between 50 and 75 ppm are linked to the appearance of two Al(IV) peaks; a broad Al(IV)-a (50–70 ppm) attributed to Al in bridging positions and a sharp Al(IV)-b (72 ppm).

The presence of Al(IV)-b (72 ppm) means that we shall observe a Q^2^(1Al)-in pairing position, which is consistent with the ^29^Si MAS NMR spectra in (Figure 13b). Such a Q^2^(1Al) in the pairing position is in agreement with the less pronounced Q^1^ peak in SS0 than in SS8 at 2 days (Figure 13).

In the octahedrally coordinated region of the spectra (between 20 and 0 ppm^38^), three bands are observed:

- At 12 ppm, there is a resonance associated with the formation of AFt phases,^35,40^ which is considerably increased by adding sodium sulfate but showing a very slight reduction in intensity between 2 and 28 days (Figure 14b). For the SS0 system, this peak is considerably shorter (than for SS8) but still noticeable and shows a decrease intensity as hydration progresses (Figure 14a). This is consistent with the evolution of ettringite shown in Figure 4 and described in Section 3.2.
- At 9/10 ppm, another sharp band is observed. LDH-type phases were found in agreement with this position in ref (41). Such a resonance is similar among the four spectra analyzed, either in terms of height or width regardless of the hydration time. It suggests that the activator’s addition does not appear to influence the formation of this phase up to 28 days of hydration. These LDHs may contain SO_4_^2–^, CO_3_^2–^, Cl^–^, NO_3_^–^, or OH^–^ in the interlayer, and these anions define the basal distance for 80 °C-synthesized hydrotalcites.^41^ In the present study, the addition of sulfates may entail that SO_4_^2–^ are present in that LDH interlayer instead of OH^–^ or CO_3_^–^ (coming from limestone in PC). This may partly explain why these LDHs are observed for SS0 (Mc/Hc) but not for SS8 in XRD (Figure 1). This is supported by Figures 9 and 11 where the presence of Ca/Al and Mg/Al LDHs is observed.
- The third peak (or shoulder) is not clearly visible. However, the change in the slope for all the curves suggests that there has to be a peak around 4 or 5 ppm. Recent evidence^42^ suggests that this resonance is most likely silicate-bridging [AlO_2_(OH)_4_]_5_^–^ sites in C-A-S-H type gels and therefore the third aluminate hydrate usually reported in that position^38^ does not exist.

## 4 Conclusions

This study evaluated the phase assemblage evolution of a blended and a hybrid alkali-activated cement (with Na_2_SO_4_ as the activator), both containing high amounts (70%) of GGBFS and PC (30%). The main hydration product forming in the blended cements was a C(-A)-S-H phase. By adding sodium sulfate to these systems, the formation of a C-A-S-H type gel highly intermixed with sulfate-rich (AFt and AFm) phases along with Ca/Al and Mg/Al LDHs was identified. The activator modified the kinetic and speed of the reaction, but it has a limited influence on the main hydration products. ^27^Al MAS NMR experiments suggested a slightly greater formation of LDHs (AFm and MgAl-rich LDHs). Due to the high content of SO_4_^2–^ in the hybrid alkali-activated cements, it is expected that this anion is in the interlayer of the MgAl-rich LDH-type phases, making their detection by XRD difficult.

The addition of the activator significantly improved the development of the microstructure at early ages (<2 days), which is usually a major challenge limiting the use of high volumes of GGBFS in blended cements. DTG and QXRD results consistently showed a lower amount of portlandite in the hybrid alkali-activated system at any given curing age, indicating an increasing DoR of PC or GGBFS up to 28 days of curing. However, similar degrees of GGBFS reaction were identified in blended and hybrid alkali-activated cements at 90 d of curing. ^27^Al and ^29^Si MAS NMR results showed that no aluminates were found in cross-linking sites. However, Al gets in the C-S-H bridging and pairing positions for the hybrid system, which was less abundant for the blended system up to 28 days. This study demonstrates the effectiveness of sodium sulfate as an accelerator of the reaction and microstructure development of cements with a high GGBFS content and low clinker factor cement.

In terms of future perspectives, the revealed densification effect identified from 2 days of curing in the hybrid alkali-sulfate activated cements is in good agreement with a better compressive strength development. This leads to a lower global warming potential per unit of compressive strength as explained in our previous studies.^2^ Such densification may have significant effects on transport properties and chemical resistance. These effects would be relevant for durability in terms of, for example, carbonation performance, chloride ingress, and (external) sulfate attack.

## Supporting Information Available

Mass balance calculations, QXRD refinement and phase identification, SEM-BSE images, and quantification of phases and 29Si MAS NMR data associated with anhydrous PC and GGBFS (PDF)
