# Supplementary material for: Phase Evolution of Hybrid Alkali Sulfate-Activated Ground-Granulated Blast Furnace Slag Cements
Source: ACS Sustain Chem Eng. 2023 Dec 1;11(49):17519–31. doi: 10.1021/acssuschemeng.3c05937 (PMC10836761; doi:10.1021/acssuschemeng.3c05937)
Supplement: Supplementary file 1 — sc3c05937_si_001.pdf [file sc3c05937_si_001.pdf]

Supporting information for:

Phase Evolution of Hybrid Alkali Sulfate Activated Ground Granulated Blast Furnace Slag Cements

Juan Manuel Etcheverry [1,*], Zengliang Yue [2], Sreejith Krishnan [2],
Yury Andres Villagran-Zaccardi [1,3], Philip Van den Heede [1], Yuvaraj Dhandapani [2], Susan Andrea Bernal [2], Nele De Belie [1*]

[1] Magnel-Vandepitte Laboratory for Structural Engineering and Building Materials, Ghent University, Technologiepark-Zwijnaarde 60, 9052 Ghent, Belgium.

[2] School of Civil Engineering, University of Leeds, Leeds LS2 9JT, United Kingdom.

[3] Sustainable Materials, Flemish Institute for Technological Research (VITO), Boeretang 200, 2400 Mol, Belgium.

*Corresponding author: email: JuanManuel.Etcheverry@UGent.be; Nele.DeBelie@UGent.be

Number of pages: 8

Number of figures: 5 [Fig. S1-S1, S1-S2, S1-S3, S1-S4 S1-S5 and S1-S6]

Number of tables: 2 [Table S1-S1 and S1-S2]

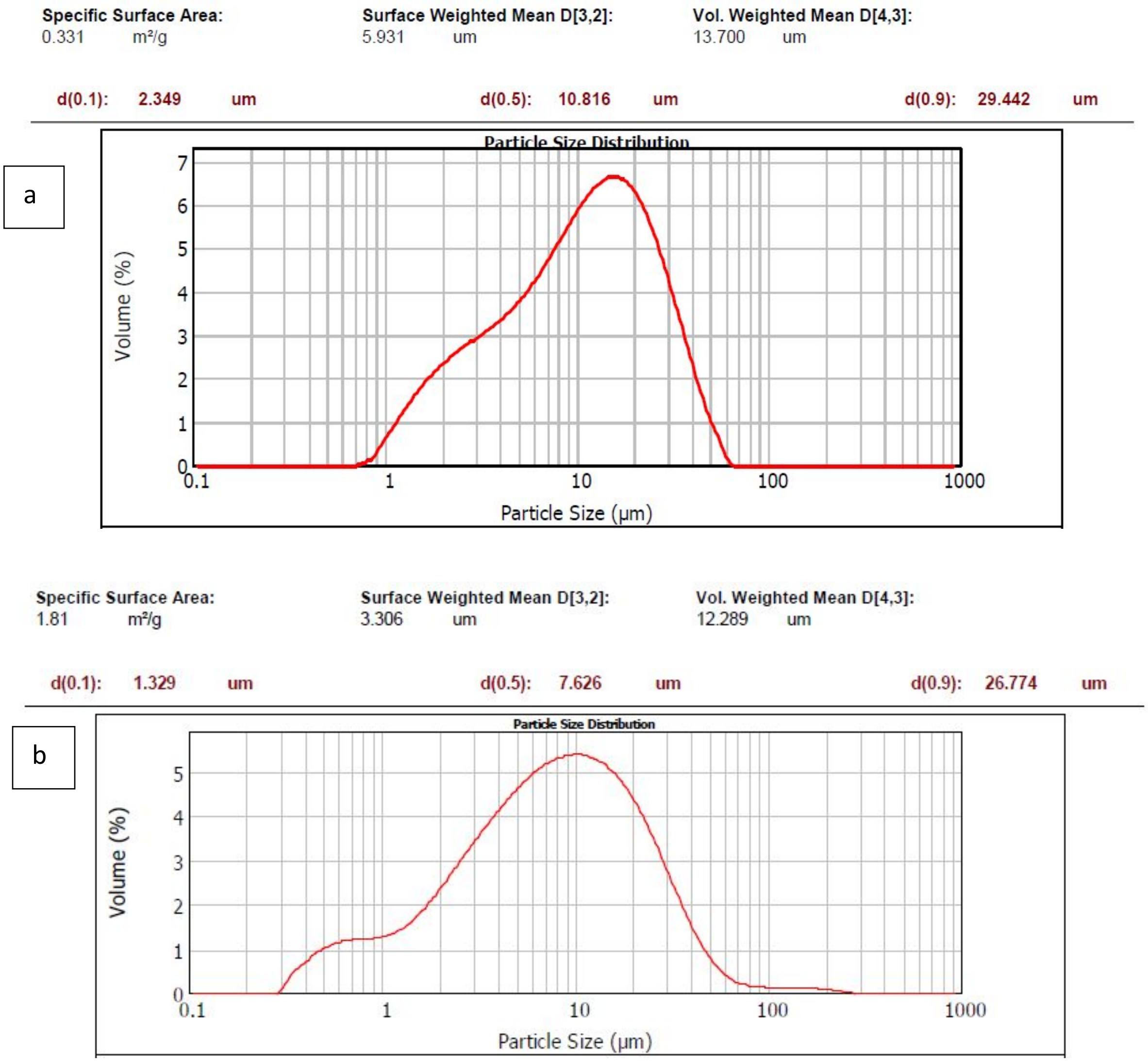

Fig. S1-S1. Particle size distribution a) CEM I and b) GGBFS determined by laser diffraction.

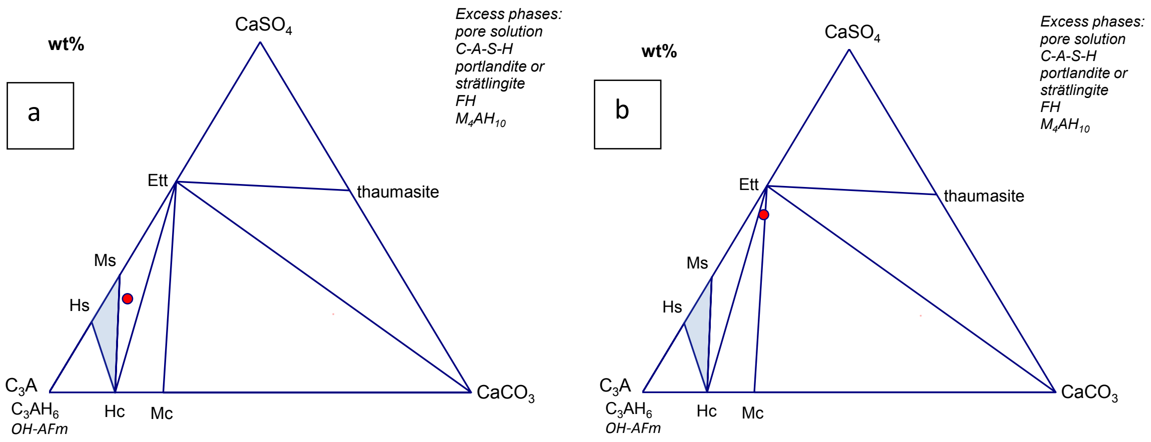

Fig. S1-S2. Mass balance calculations conducted according to [1] for SS0 a) and SS8 b).

QXRD analysis: additional information concerning refinement and phase identification:

For the refinement, global parameters were refined for specimen displacement, and background (Chebyshev Polynomial with 6 coefficients), and phase-specific parameters were refined for scale factors, lattice parameters (with constraint limits ±1% of reference values), peak shape with the fundamental parameter approach for crystalline phases and Pseudo-Voigt approach for C(-A)-S-H and GGBFS, preferred orientation correction for appropriate phases (portlandite, ettringite), and fixed atomic positions and displacement. Phase information for the reported phases is presented in Table S1-S1. The chemical bound water determined from TGA measurements was taken into consideration to "scale" the volumetric variations due to the hydration process at different ages.

Table S1-S1. Phases information for QXRD.

Phase name	Chemical formula	Pattern reference
Alite	Ca_3SiO_5	ICSD 94742
Belite	Ca_2SiO_4	ICSD 81096
Ferrite	Ca_2AlFeO_5	ICSD 9197
Ettringite	$Ca_6Al_2(SO_4)_3(OH)_{12}(H_2O)_{25.68}$	COD 9011103
Portlandite	$Ca(OH)_2$	ICSD 202229

Hemicarbonate	$Ca_4Al_2(OH)_{12}(OH)(CO_3)_{0.5}(H_2O)_5$	COD 2105252
Monocarbonate	$Ca_4Al_2(OH)_{12}(OH)(CO_3)_{0.5}(H_2O)_5$	COD 2007668
Strätlingite	$Ca_2Al((AlSi)_{1.11}O_2)(OH)_{12}(H_2O)_{2.25}$	ICSD 69413
Calcite	$CaCO_3$	ICSD 73446
Zincite	ZnO	ICSD 38222
C(-A)-S-H	Model profile of diffuse scattering contribution with peaks at 29.50, 34.26 and 50.90 ° 2θ, respective relative intensities of 1, 0.74, and 0.93 adjusted by scale and a lorentzian peak broadening parameter	
GGBFS	Model profile of diffuse scattering contribution with peak at 29.80 ° 2θ adjusted by scale and a lorentzian peak broadening parameter	

Additional information about SEM-BSE images and quantification of phases:

For each series, at least 30 BSE images were evaluated at ages of 2, 7, and 28 days for the two systems studied. A Matlab code was utilized for the phase quantification based on the grey scale histogram of the 30 images as described in [13]. The threshold values were identified at the points of local minimal frequency before and after each peak of the histograms. The challenge of this quantification approach resides in the fact that GGBFS and portlandite (CH) have almost the same grey level. Normally, the quantification needs to consider also the morphology of the phases (more irregular and dendritic for CH compared to sharper angular particles for GGBFS). See the example in Fig S1-S4 showing some areas that might be attributable to portlandite, but are still partially identified as GGBFS. The binders in this study (whether activated or not) contained low amounts of PC, which resulted in limited contents of portlandite in the hydrated pastes (<4 wt.%, as confirmed by TGA and QXRD below). In addition, the lack of detection of very fine, unreacted GGBFS

particles (an inherent shortcoming of the SEM method and its configuration) somewhat mitigates the effect of some portlandite being characterised as GGBFS. After evaluating the limited impact of these factors for the particular binders under study, it was pondered that the introduction of additional correction procedures based on the shape of grains would introduce greater inaccuracy than considering only the greyscale values. From this reasoning, any prospective distortion in the quantification of unreacted GGBFS is expected to be minimal.

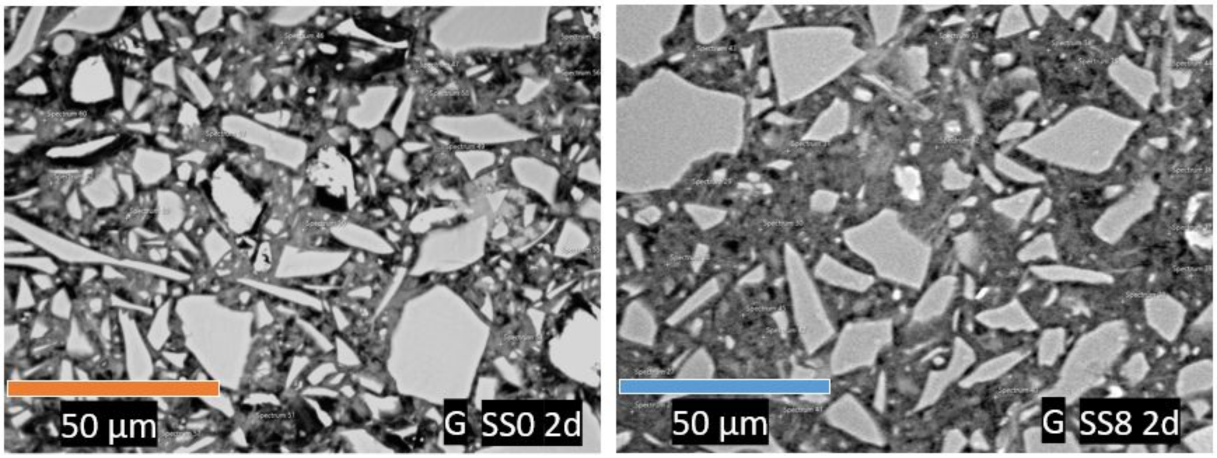

Fig. S1-S3. Point selection SEM-EDS analysis at 2 days of hydration for SS0 and SS8.

Table. S1-S. QXRD anhydrous cement (Wt% Rietveld)

Alite	60.44
Belite	8.92
Aluminate cubic	2.10
Aluminate orthorhombic	5.54
Ferrite	11.66
Anhydrite	5.09
Calcite	2.10
Others	4.15

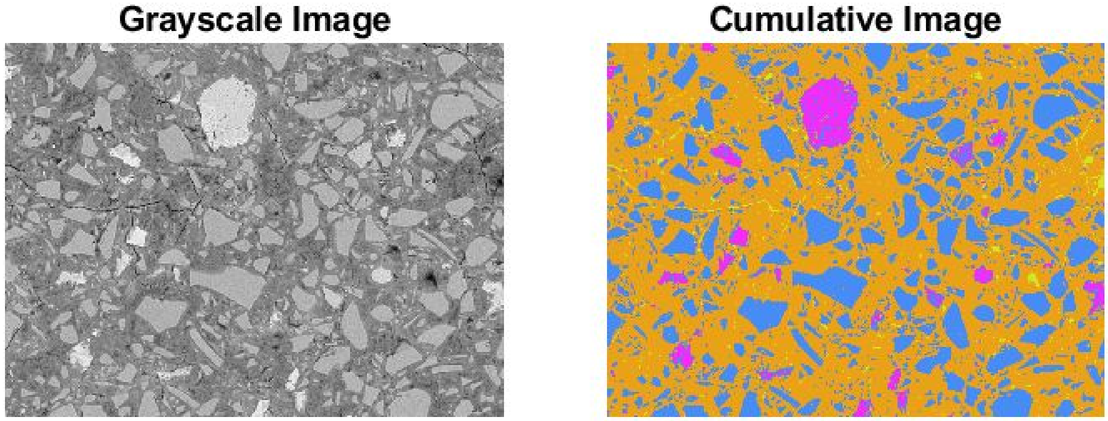
Grayscale Image
Cumulative Image

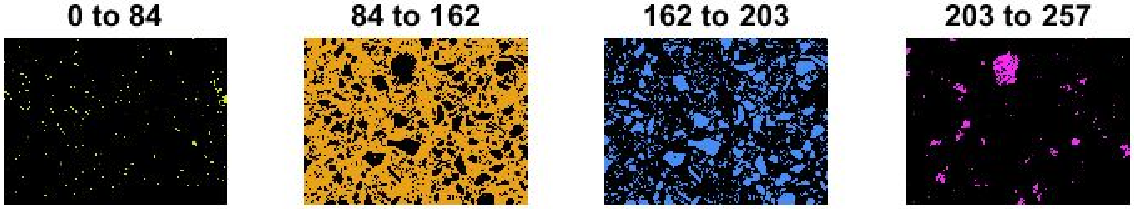
0 to 84
84 to 162
162 to 203
203 to 257

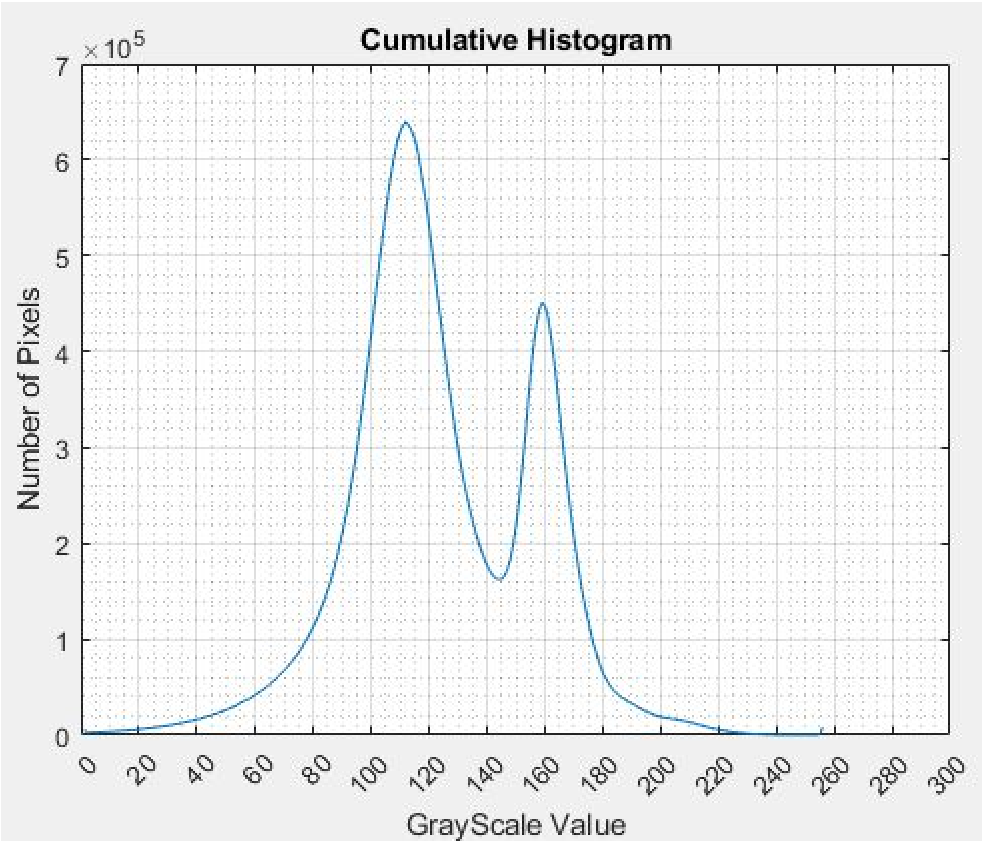
Cumulative Histogram
×10^5
Number of Pixels
GrayScale Value
0
1
2
3
4
5
6
7
0
20
40
60
80
100
120
140
160
180
200
220
240
260
280
300

Fig. S1-S4. Example of the greyscale image and the resulting segmentation from SEM-BSE. Below, the quantification of each greyscale range detected. Porosity in yellow, C-S-H in orange, Unreacted slag particles in blue and Unreacted PC grains in pink. Histogram of frequencies for the selection of greyscale points

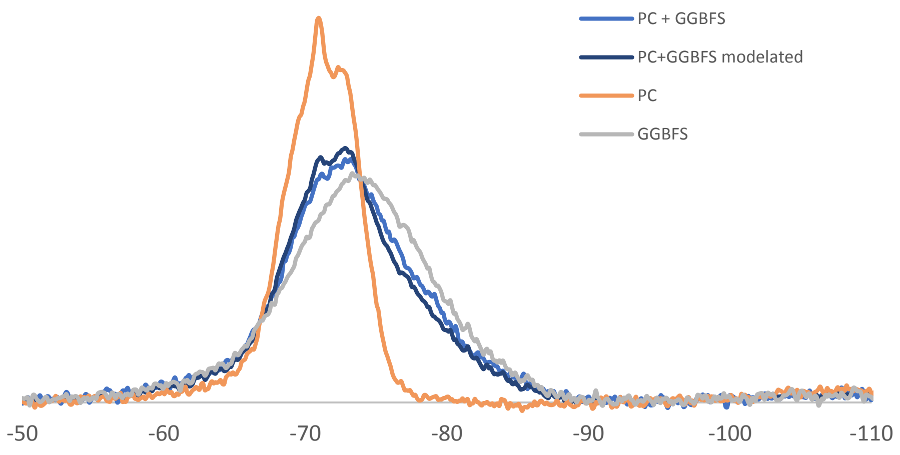

Fig. S1-S5. ^{29}Si MAS NMR on anhydrous materials. Horizontal axis in ppm.

<u>Additional information Solid-state 27Al and 29Si MAS NMR:</u>

Sufficient recycle delays during NMR data acquisition to allow for complete relaxation is important. A recycle delay of 40 s for 29Si MAS NMR measurements might be too short to obtain quantitative information about the abundance of silicate species. For the magnetic field used in this study (9.4 T), and the large rotors used in the experiments (7 mm) the testing parameters selected are considered appropriate for the identification of C-S-H or C-A-S-H phases, which are the main Si-rich phases expected to be formed in these systems. The FID (free induction decay) plot for one of the samples evaluated (**Fig. S1-S6**) shows the signal is stable over time and a recycled delay of 40 s seemed sufficient for obtaining quantitative information and therefore it has been considered that the analysis we have reported in the study is correct. It is worth noting that other studies of cements performed at a similar magnetic field and sample quantity/ rotor size [2] have been conducted at even lower recycled delays (e.g. 30 s) enabling clear identification of bands associated with clinker phases and reaction products forming. From the data given in Section II of the paper [2], it can be calculated that the flip angle in that study was approx. 43°; thus, assuming the same

chemical system, a much shorter recycle delay than for a 90° pulse (which has been used in the present study) would be sufficient. In addition, the same relaxation behaviour cannot be assumed based only on the fact that [2] studied C-S-H. Thus, it might be still unproven that the NMR measurements of the present study were fully quantitative. Nevertheless, NMR results presented in Section 3.5 of the current study are internally consistent and consistent with the EDS results, which may be taken as an indication that the measurements were essentially quantitative.

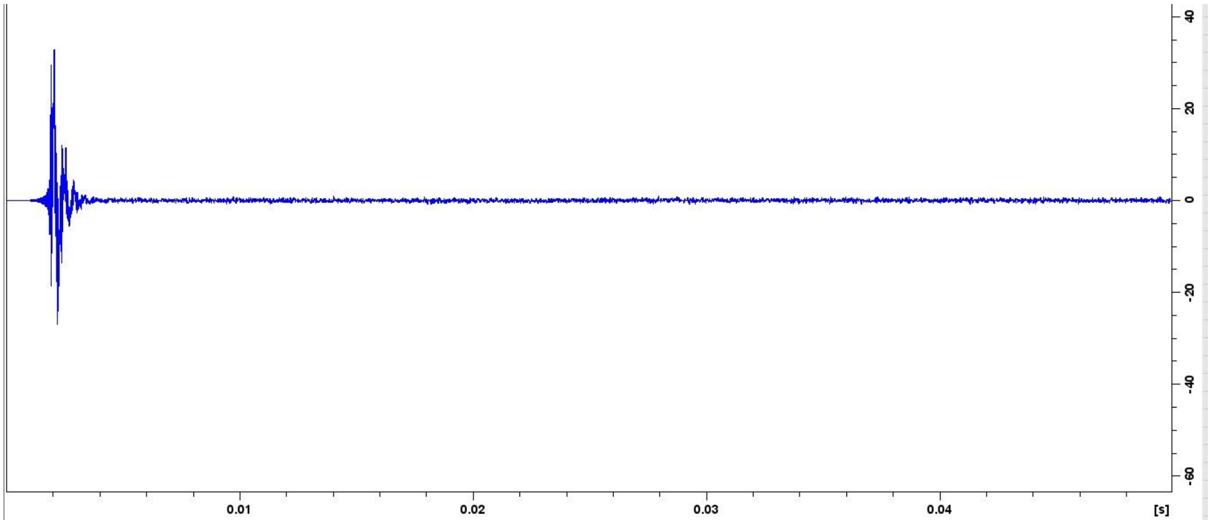

Fig. S1-S6. FID (free induction decay) plot for one of the samples evaluated in this study.

References:

[1] D. Herfort and B. Lothenbach, “Calculation of ternary diagrams by mass balance.” EMPA, p. Available online 29.06.2023, 2016. [Online]. Available: https://www.empa.ch/web/s308/ternary-diagram

[2] S. Hydrate, C. S. H. P. Resulting, and C. Hydration, “The Effect of Alkali Ions on the Incorporation of Aluminum in the Calcium Silicate Hydrate (C – S – H) Phase Resulting from Portland Cement Hydration Studied by 29 Si MAS NMR,” vol. 656, pp. 651–656, 2013, doi: 10.1111/jace.12024.
